# Co-Delivery of Ylang Ylang Oil of *Cananga odorata* and Oxaliplatin Using Intelligent pH-Sensitive Lipid-Based Nanovesicles for the Effective Treatment of Triple-Negative Breast Cancer

**DOI:** 10.3390/ijms24098392

**Published:** 2023-05-07

**Authors:** Nada K. Sedky, Nour M. Abdel-Kader, Marwa Y. Issa, Manal M. M. Abdelhady, Samir N. Shamma, Udo Bakowsky, Sherif Ashraf Fahmy

**Affiliations:** 1 Department of Biochemistry, School of Life and Medical Sciences, University of Hertfordshire Hosted by Global Academic Foundation, R5 New Garden City, New Administrative Capital, Cairo 11835, Egypt; 2 Department of Biochemistry, Faculty of Science, Ain Shams University, Cairo 11566, Egypt; 3 Department of Pharmacognosy, Faculty of Pharmacy, Cairo University, Kasr El-Aini Street, Cairo 11562, Egypt; 4 Clinical Pharmacy Department, Faculty of Pharmacy, Badr University, Cairo 11829, Egypt; 5 Institute of Global Health and Human Ecology, School of Sciences & Engineering, The American University in Cairo, AUC Avenue, P.O. Box 74, New Cairo 11835, Egypt; 6 Department of Pharmaceutics and Biopharmaceutics, University of Marburg, Robert-Koch-Str. 4, 35037 Marburg, Germany; 7 Department of Chemistry, School of Life and Medical Sciences, University of Hertfordshire Hosted by Global Academic Foundation, R5 New Garden City, New Administrative Capital, Cairo 11835, Egypt

**Keywords:** essential oils, platinum-based anticancer drugs, oxaliplatin, niosomes, cytotoxicity, invasive breast cancer, apoptosis, intrinsic pathway

## Abstract

Smart pH-responsive niosomes loaded with either Oxaliplatin (Ox), Ylang ylang essential oil (Y-oil), or co-loaded with both compounds (Ox-Y) (Ox@NSs, Y@NSs, and Ox-Y@NSs, respectively) were formulated utilizing the thin film method. The developed nanocontainers had a spherical morphology with mean particle sizes lower than 170 nm and showed negative surface charges, high entrapment efficiencies, and a pH-dependent release over 24 h. The prepared pH-responsive niosomes’ cytotoxicity was tested against the invasive triple-negative breast cancer (MDA-MB-231) cells, compared to free OX and Y-oil. All niosomal formulations loaded with Ox and/or Y-oil significantly improved cytotoxic activity relative to their free counterparts. The Ox-Y@NSs demonstrated the lowest IC50 (0.0002 µg/mL) when compared to Ox@NSs (0.006 µg/mL) and Y@NSs (18.39 µg/mL) or unloaded Ox (0.05 µg/mL) and Y-oil (29.01 µg/mL)**.** In addition, the percentages of the MDA-MB-231 cell population in the late apoptotic and necrotic quartiles were profoundly higher in cells treated with the smart Ox-Y@NSs (8.38% and 5.06%) than those exposed to free Ox (7.33% and 1.93%) or Y-oil (2.3% and 2.13%) treatments. Gene expression analysis and protein assays were performed to provide extra elucidation regarding the molecular mechanism by which the prepared pH-sensitive niosomes induce apoptosis. Ox-Y@NSs significantly induced the gene expression of the apoptotic markers Tp53, Bax, and Caspase-7, while downregulating the antiapoptotic Bcl2. As such, Ox-Y@NSs are shown to activate the intrinsic pathway of apoptosis. Moreover, the protein assay ascertained the apoptotic effects of Ox-Y@NSs, generating a 4-fold increase in the relative protein quantity of the late apoptotic marker Caspase-7. Our findings suggest that combining natural essential oil with synthetic platinum-based drugs in pH-responsive nanovesicles is a promising approach to breast cancer therapy.

## 1. Introduction

Breast cancer is one of the most common causes of death in females worldwide [1]. Oxaliplatin (Ox) is a third-generation platinum-based chemotherapeutic agent that promotes cytotoxicity against breast cancer via inducing reactive oxygen species (ROS) and, hence, increasing oxidative stress [2,3,4]. In addition, Ox binds covalently to the cancer cells’ DNA, causing apoptosis and cell cycle arrest [5,6]. Through its potent anticancer effects, Ox causes several toxic effects, including neurological, hematological, and gastrointestinal toxicities [7]. These adverse effects can lead to treatment cessation [8] and are the main dose-limiting toxicities in patients treated with oxaliplatin [9].

Natural essential oils (EOs) have widely been used in various wellness and healthcare products [10]. Essential oils are a complex mixture of volatile lipophilic molecules [11]. Their biochemical effects range from their use in the plant defense mechanism against different infections and parasites [12] to their utilization in several medicinal approaches, including antibacterial, anti-inflammatory [13], antiviral [14], and anticancer activities [15]. Cancer therapy utilizing EOs, or their ingredients, has gained much interest lately. Numerous in vitro/in vivo investigations have demonstrated their anticancer effect in various tumor cell lines and animal models [16].

The use of essential oils in conjunction with other chemotherapeutic drugs, particularly platinum-based drugs, has shown several synergistic effects. The combination of nanoemulsion essential oil (from *Teucrium polium* L.) with Ox has recently been investigated in the treatment of colon cancer cell lines (HCT116 wild-type and HT-29 with p53 mutant-type), where the apoptotic effect of Ox was significantly elevated through a ROS-induced mitochondrial dysfunction mechanism [17]. In addition, the combination of cisplatin and *T. ostenii* essential oil extracts significantly affected the viability, adhesion, migration, and clonogenic ability of the human cervical carcinoma cell line, SiHa (HPV 16-positive) [18]. The ability of *Curcuma longa* EO extracts to induce a therapeutic effect resulted in the re-sensitization of ovarian cancer chemo-resistant cell lines to cisplatin or paclitaxel chemotherapeutic agents. The combined treatment also reduced the required drug concentration as it enhanced the target drug’s cytotoxicity and consequently limited its adverse side effects [19].

Ylang ylang (*Cananga odorata*) is a naturally growing plant in Asian nations, with a pleasant floral aroma similar to jasmine [20]. Its essential oil (Y-oil) has long been used in the perfume industry, aromatherapy, and food sectors [21]. Y-oil has been reported to possess antiviral [22], antibacterial [23,24], antioxidant [25], and soothing properties [26]. Several factors can affect the essential oil composition, including geographical origin, climate, and seasonal variations [27,28]. Despite the broad interest in studying essential oils’ anticancer effects, minimal studies have been conducted on Y-oil to elucidate its primary anticancer mode of action. One reported study highlighted the antitumor activity of a bioactive ingredient, β-caryophyllene (a terpene presents in many plants, including Ylang ylang), against prostate and breast cancer [28,29]. In addition, β-caryophyllene induces apoptosis in lymphoma cell lines [29] and has demonstrated cytotoxic activity over a wide range of cancer cell lines [30]. Liriodenine (an oxoaporphine alkaloid) is another bioactive nonvolatile ingredient in the seeds of *C. odorata*, which showed a powerful inhibition of topoisomerase II in vitro and in vivo, accounting for its cytotoxic and antineoplastic effect [31]. Similarly, both Cryptomeridiol,11-α-L-rhamnoside and γ-eudesmol secondary metabolites exhibited potent cytotoxic activity against Hep G2 and Hep 2,2,15 cell lines [21,32].

Although natural extracts have potential anticancer activities, their clinical applications are limited owing to their instability, hydrophobicity, poor bioavailability, and volatility. Thus, nanoformulation was invented to overcome the drawbacks of the clinical use of natural essential oils [33]. Recent advances in nanomedicine have enabled the delivery of various bioactive and natural compounds, boosted their therapeutic efficiencies and overcame their physicochemical and biological limitations [34,35,36,37].

In particular, niosomes represent a group of amphiphilic vesicular nanoparticles, which are formed by self-assembling nonionic surfactants and lipids. They have shown improved physicochemical and therapeutic durability compared to liposomes [38]. For instance, niosomes overcome some of the shortcomings of using liposomes, such as low stability and challenging large-scale production. In addition, niosomes have gained much interest as a promising drug delivery system owing to their long-term stability, ability to increase solubility, and cellular uptake of various therapeutically active cargos [39]. Moreover, niosomes have been reported to ameliorate the pharmacokinetics and biodistribution profiles of different drugs and boost their bioavailability. In addition, pH-responsive nanocarriers have been developed to trigger the release of the loaded drugs preferentially in the acidic microenvironment of cancer cells (Cancerous cells pH = 5.7 late endosomes and lysosomes pH = 4.5–5.5) [40]. In this regard, pH-sensitive niosomes have been fabricated using cholesteryl hemisuccinate (CHEMS), a pH-responsive agent [41]. CHEMS is a cholesterol ester that self-assembles in a neutral medium, forming intact nanovesicles under physiological conditions (pH 7.4), whereas it disassembles in the acidic microenvironment of cancer cells. Consequently, these smart lipid-based nanovesicles can boost the anticancer activity of the loaded drugs and reduce their adverse effects [41].

This work explores Ox and Y-oil‘s synergistic anticancer activities when co-loaded into pH-responsive niosomes. In this regard, the essential oils of Y-oil were obtained using a green approach via the hydrodistillation of fresh, fully opened Ylang ylang flowers (*Cananga odorata*, forma *genuine*), and their constituents were chemically identified via gas chromatography–mass spectrometry (GC–MS). Then, the extracted Y-oil was co-loaded with Ox into pH-responsive niosomes (Ox-Y@NSs) using the thin film approach. The prepared niosomes were then characterized, and the entrapment efficiency and the in vitro release percent (at two different pH values) were evaluated. The cytotoxic activity of the synthesized niosomes, as well as the free Ox- and Y-oil, were tested against MDA-MB-231 cells. The estimated IC50s were used for further apoptosis and gene expression analyses of the tumor suppressor gene (Tp53), the apoptotic genes (Bax and Caspase-7), and the antiapoptotic Bcl-2 gene. Protein determination via western blotting was also performed for the late apoptotic marker (Caspase-7).

## 2. Results and Discussion 

### 2.1. GC–MS Peaks Identification in Ylang Ylang Flowers Essential Oil

**Figure 1:**
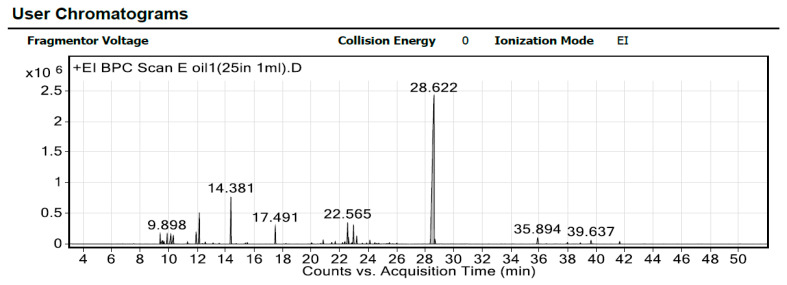
GC–MS representative chromatogram of Y-oil (*Cananga Odorata* Hook. F. and Thomson).

The volatile content in Y-oil was extracted using a green approach and was assessed, aiming to explore the chemical constituents responsible for its anticancer activity against triple-negative breast cancer cell lines. The identified compounds and their relative proportions are listed according to their elution order on the HP-5MS UI column (Table 1, Figure 1). 

GC–MS analysis of Y-oil resulted in the identification of 52 volatile components categorized into six major classes, namely alcohols, esters, monoterpene hydrocarbons, phenols/ethers, phthalate esters, and sesquiterpene hydrocarbons. This is in addition to four other classes, aldehydes, glycerols, ketones, and sesquiterpenoid oxides, each constituting one compound only. Several metabolites were reported as representative components of Y-oil, including diethyl phthalate, cadinene, benzyl acetate, β-linalool, thujopsene, and linalyl anthranilate, as detailed in the supplementary materials. Our findings aligned with a previous study conducted by Tambe et al. [42] and indicated the successful extraction of Y-oil.

The GC–MS analysis revealed that the Y-oil’s most abundant class of compounds is phthalate esters, comprising diethyl phthalate ester (29.08%). The second most abundant class of compound is sesquiterpenes (27.33%), comprising *α*-Gurjunene (11.04%), thujopsene (6.83%), aromandendrene (2.45%), and γ-gurjunene (1.08%).

### 2.2. Characterization of the pH-Responsive Niosomes

The dynamic light scattering technique was utilized to study the mean particle sizes and polydispersity index (PDI) of the prepared pH-responsive niosomes (blank NSs, Y@NSs, Ox@NSs, and Ox-Y@NSs) at 25 °C, as presented in Table 2. All niosmal formulations showed uniform sizes and monodispersity, with PDI values lower than 0.2. The mean particle size of blank NSs was found to be 94.16 ± 19.0, which was increased to 129.72 ± 15.5, 126.93 ± 12.5 and 159.34 ± 16.9 after the loading of Y-oil, OX, and Ox-Y, respectively. This suggests the efficient incorporation of the drugs into the pH-responsive niosomes [43]. All prepared niosomes exhibited sizes smaller than 160 nm, thus empowering the passive accumulation of the pH-responsive niosomes, specifically in tumor cells [3,44,45]. 

**Figure 2:**
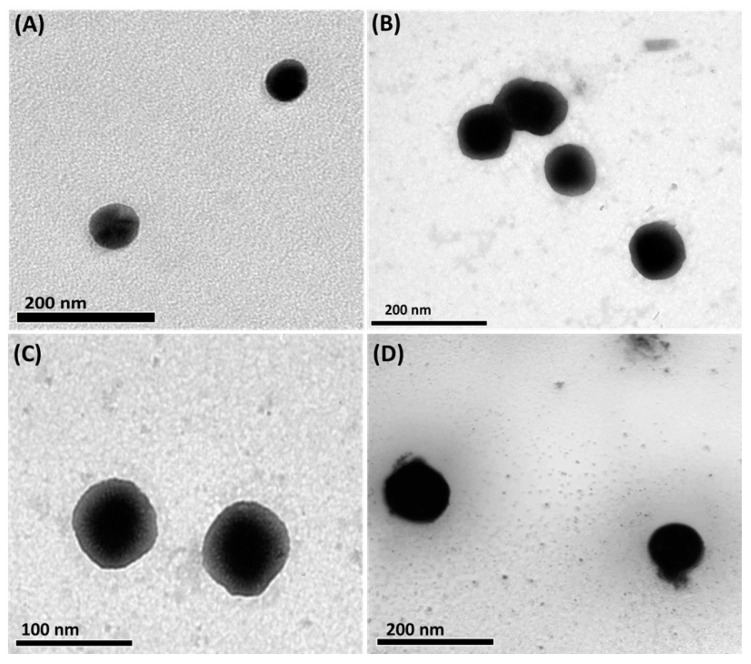
TEM images of (**A**) blank NSs, (**B**) Y@NSs, (**C**) Ox@NSs, and (**D**) Ox-Y@NSs, demonstrating the spherical morphology of the prepared pH-responsive niosomes.

As presented in Table 2, the zeta potential values of the developed pH-responsive niosomes were −12.37 ± 1.7, −14.65 ± 1.5, −10.53 ± 1.1, and −13.31 ± 1.9 mV for plain NSs, Y@NSs, Ox@NSs, and Ox-Y@NSs, respectively. The negatively charged niosomal nanovesicles could repel each other, increasing the shelf life stability by reducing aggregation [46]. Previous studies reported higher entrapment efficiency percent (EE %) values for niosomes prepared using the thin film hydration method compared to those formulated via other methods [47]. This was evident from the current findings, where the EE% values of Y@NSs, Ox@NSs, and Ox-Y@NSs were above 80%, indicating the ability of the pH-responsive niosomes to entrap high concentrations of either Y-oil, Ox, or Ox-Y, thus ensuring drug bioavailability and enhanced therapeutic activity (Table 2). The shapes of the prepared pH-responsive niosomes were assessed via TEM analysis. As illustrated in Figure 2, all prepared niosomes exhibited a rounded morphology with smooth surfaces and negligible aggregations. In addition, our findings revealed that the dual loading of Ox-Y into niosomes did not affect their spherical morphology. 

### 2.3. In Vitro Release Study

**Figure 3:**
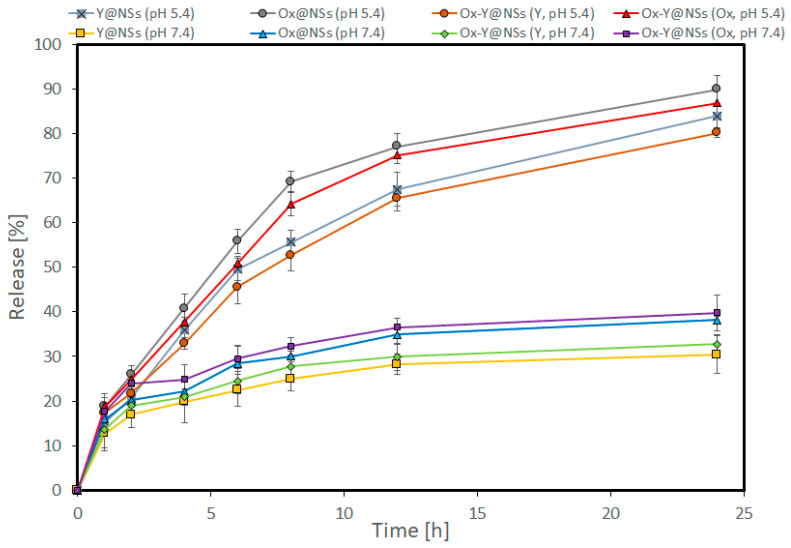
Release [%] of Y-oil, Ox, and Ox-Y from pH-responsive Y@NSs, Ox@NSs, and Ox-Y@NSs into phosphate buffer (pH 7.4) and acetate buffer (pH 5.5.4). Data are presented as mean ± SD; *n* = 3.

The dialysis bag method investigated the in vitro release percent of Y-oil, Ox, and Ox-Y from the different pH-responsive CHEMS niosomal formulations (Figure 3). This study was conducted using two different pH environments simulating the actual conditions in cancer cells (pH 5.4) and the physiological body fluids (pH 7.4) in order to evaluate the effect of pH sensitivity on the drug rates of the drug release. It has been shown that integrating CHEMS in the niosomes significantly increases the release rates of the payloads in acidic pH (5.4). In this study, the release rates of Y-oil, Ox, and Ox-Y from Y@NSs, Ox@NSs, and Ox-Y@NSs at pH 5.4 were significantly faster (83, 89, 80, and 87%, respectively) than at pH 7.4 (30, 38, 32 and 40%, respectively) within 24 h (*p* < 0.05). Our findings revealed that the diffusion of the loaded drugs out of the niosomal formulations is sensitive to the acidic pH, suggesting a preferential release in tumor tissues. Eventually, this will improve the loaded drugs’ therapeutic effects on the cancerous cells without impacting the normal ones.

### 2.4. Cytotoxicity (SRB Assay)

MDA-MB-231 cells were treated for 72 h with eight increasing concentrations (ranging from 0.0001–1000 μg/mL) of NSs, Y-oil, Ox, Y@NSs, Ox@NSs, and Ox-Y@NSs, in addition to blank pH-responsive NSs; after that, the cellular viability was evaluated using the SRB assay.

**Figure 4:**
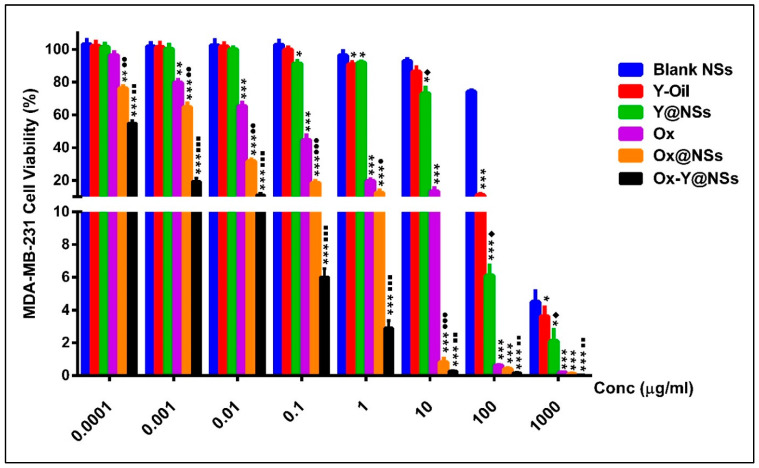
The cell viability of MDA-MB-231 cells after exposure to the different agents for 72 h. Data are displayed as the mean of three individual runs ± SD. The symbol (*) refers to the statistical significance of the Blank NSs-treated cells (Control). The symbol (♦) refers to the statistical significance of Y-oil treated cells. The symbol (●) refers to the statistical significance of Ox-treated cells. The symbol (■) refers to the statistical significance of Ox@NSs. The presence of any symbol once only indicates significance with a *p*-value ≤ 0.05. Any symbol repeated twice indicates significance with a *p*-value ≤ 0.01, and a symbol repeated thrice indicates a *p*-value ≤ 0.001.

Figure 4 and Table 3 show that free Y-oil had an IC_50_ value of 29.01 µg/mL. Interestingly, when Y-oil was loaded into NSs, the formulation exhibited a lower IC_50_ value of 18.39 µg/mL (a two-fold increase in cytotoxicity compared to free Y-oil). On the other hand, Ox displayed an IC_50_ value of 0.05 µg/mL, which decreased to 0.006 µg/mL when loaded into NSs (an eight-fold increase compared to free Ox). Importantly, when Y-oil and Ox were co-loaded into NSs, the formulation showed the lowest IC50 value, reaching 0.0002 µg/mL (a thirty-fold increase in cytotoxicity compared to Ox@NSs). Finally, and to show that the blank NSs did not cause the cytotoxic activity of the NSs encapsulating Y-oil and Ox, we tested the effect of the blank NSs on MDA-MB-231 cellular viability. Our findings showed that none of the investigated concentrations of the blank NSs showed any significant cytotoxicity on MDA-MB-231 cellular viability. Thus, the cytotoxicity of Y-oil and Ox co-loaded in NSs was not attributed to any cytotoxic activities of the blank NSs. To assess the enhanced anticancer effect when combining Y-oil and Ox into pH-responsive NSs (Ox-Y@NSs), we assessed the CI depending on the IC_50_ values of each of them alone and combined. Our findings showed that the CI was 0.02, suggesting synergism between both Y-oil and Ox; this is considering that CI < 0.8 shows synergism, a CI ranging from 0.8 to 1.0 suggests an additive effect, and CI > 1 shows an antagonistic effect [37,48].

Previous studies have reported the antitumor activity of Y-oil against hepatocarcinoma [21], MCF-7 and MDA-MB-231 human breast tumor cell lines [49,50], and Ehrlich Ascites Carcinoma-treated mice model [51]. The anticancer activities of Y-oil are attributed to the high contents of diethyl phthalate ester (29.08%) and sesquiterpenes hydrocarbons (27.33%), as identified by the GC–MS analysis. Both compounds were reported to generate DNA damage and induce apoptosis, promoting cytotoxicity against different cancer cell lines, including MCF-7 breast cancer cells. [49,52]. Moreover, the cytotoxic activity of Ox has been reported against triple-negative MDA-MB-231 cells [53]. The pH-responsive behavior of the formulated NSs allows the selective release of the payloads in the acidic tumor microenvironment and their ability to accumulate passively into MDA-MB-231 cells via the enhanced permeability and retention (EPR) effect, thus explaining the enhanced cytotoxicity of all developed NSs relative to their free counterparts. It is worth mentioning that no previous studies, to the best of our knowledge, have reported the dual loading of Y-oil and Ox into pH-responsive NSs and the resulting boosted anticancer effect against triple-negative breast cancer. Since Ox-Y@NSs exhibited the highest cytotoxicity compared to both Y@NSs and Ox@NSs, it was selected for further mechanistic assays.

### 2.5. Apoptosis Assay

**Figure 5:**
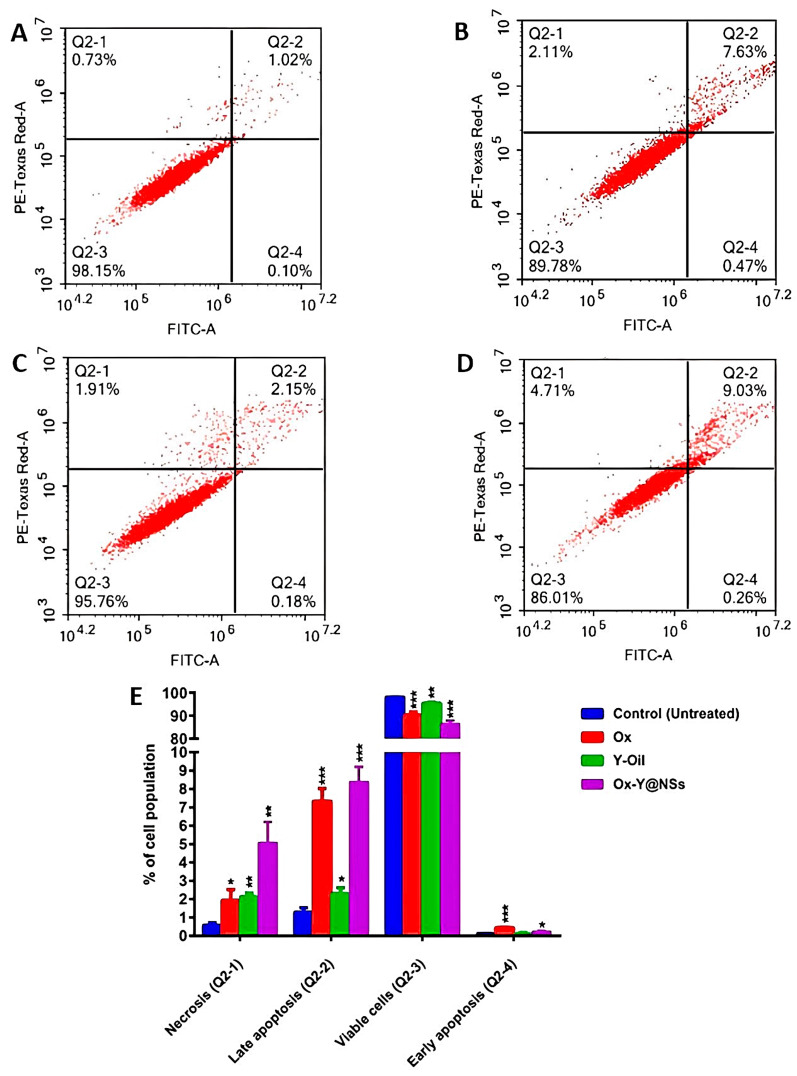
A display of the apoptosis assay results in MDA-MB-231 cells when incubated for two consecutive days with the tested compounds (Ox, Y-Oil, and Ox-Y@NSs). Cytograms presenting annexin-V/Propidium Iodide-stained untreated MDA-MB231 cells as negative control (**A**), cells treated with Ox (**B**), cells treated with Y-Oil (**C**), cells treated with Ox-Y@NSs, (**D**) and analysis of the four quartiles resulting from the apoptosis assay (**E**). Quadrant charts show Q2-1 (necrotic cells, AV-/PI+), Q2-2 (late apoptotic cells, AV + /PI+), Q2-3 (normal cells, AV-/PI-) and Q2-4 (early apoptotic cells, AV + /PI-). Data are the average of three individual experiments ± standard deviation (SD). ***, ** and * refer to significant differences from the control, where *p*-values ≤ 0.001, ≤0.01 and ≤0.05, respectively.

MDA-MB-231 cancer cells were treated with the corresponding IC_50_ concentrations of Ox, Y-oil, and Ox-Y@NSs for 48 h. Non-treated MDA-MB-231 cells were deployed as the negative control, and the results are displayed in Table 4 and Figure 5. It was noticed that treatment with Ox and Ox-Y@NSs resulted in a significant increase in cell population in the early apoptosis, late apoptosis, and necrosis quartiles compared to non-treated cells. Treatment with Y-oil solely caused a significant increase in late apoptosis and necrosis compared to non-treated cells. In addition, treatment with Ox, Y-oil, and Ox-Y@NSs caused a significant decrease in the viable cells’ quartile. It is noteworthy to mention that, in the late apoptosis and necrosis stages, Ox-Y@NSs treatment brought about the highest cell population percentage (8.38 ± 0.83% and 5.06 ± 1.14%) relative to that of the Ox treatment (7.33 ± 0.7% and 1.93 ± 0.59%) and Y-oil treatment (2.3 ± 0.32% and 2.13 ± 0.19%). Amongst all the tested treatments, Ox-Y@NSs produced the most observed decline in the viable cells’ quartile, reinforcing the cytotoxicity findings and providing further support for the outstanding potency of such a formula as an anticancer agent. Previous studies have reported the apoptotic effects of Ox on cancer cells [53,54]. Exposure to Ox for 12 to 24 h was shown to induce moderate and late apoptosis among four different cancer cell lines (colon adenocarcinoma (HT29), estrogen-dependent breast cancer (MCF7), cervical cancer cells (Hela) and lung adenocarcinoma (A549)) [54].

On the other hand, little knowledge exists regarding the apoptotic activity of Y-oil. One study reported that the capabilities of Y-oil significantly rose in the apoptotic cells’ fraction in the double staining assay of epithelial cell lines isolated from a pancreatic tumor (MIA PaCa-2 Cells) [55]. The current study is the first to describe the apoptotic profile of Y-oil on triple-negative MDA-MB-231 cells and to show its importance for treating highly aggressive, poorly differentiated triple-negative breast cancer. Moreover, the formulated Ox-Y@NSs combining Y-oil and Ox maximized the apoptotic effects at much lower doses than free Y-oil and Ox. In addition to the cytotoxicity results, the apoptotic profile reveals the potential of Ox-Y@NSs as a potential anticancer agent that surpasses the approved drug Ox and the natural Y-oil for treating the invasive MDA-MB-231. 

These results strongly indicate that the co-loading of Y-oil and Ox into pH-responsive NSs exacerbates their apoptotic killing effects and are consistent with the cell viability assay’s findings. In addition, the pH-responsive formulation augments the entry of NSs through the tumor cellular membrane, releasing its cargo in the acidic microenvironment while preventing its payloads from releasing immaturely at the physiological pH before entering the cancer cell.

### 2.6. RT-qPCR

**Figure 6:**
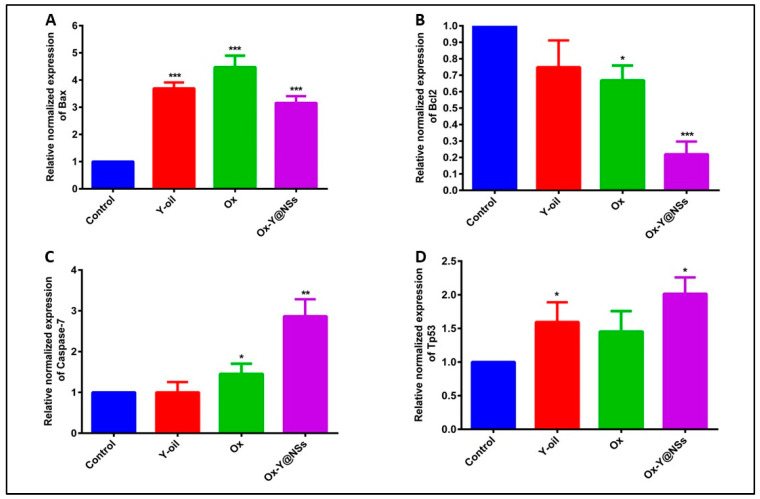
The relative normalized gene expression of Bax (**A**), Bcl2 (**B**), Caspase-7 (**C**), and TP53 (**D**) after the exposure of MDA-MB-231cells to the test compounds (Y, Ox, and Ox-Y@NSs). Each value represents three replicates. β-actin gene was selected as the housekeeping gene and was implemented in data normalization in the RT-qPCR assay. The relative normalized gene expression was calculated using the 2^−ΔΔCt^ method. ***, ** and * refer to significant differences from the control, where *p*-values ≤ 0.001, ≤0.01 and ≤0.05, respectively.

RT-qPCR was performed to determine the relative normalized gene expression of Bax, Bcl2, Caspase-7, and Tp53 genes in MDA-MB-231 cancer cells when pre-treated for 48 h with the test compounds (Y-oil, Ox, and Ox-Y@NSs) at the corresponding IC_50_ concentrations. Our study aimed to investigate the effects of the three selected compounds on the intrinsic mitochondrial pathway of apoptosis. Previous studies have reported the ability of Tp53 to induce the pro-apoptotic protein, Bax, which is implicated in the intrinsic pathway of apoptosis [56,57]. Our results displayed a considerable increase in Bax expression upon treatment with the three investigated compounds. The expression of Bax increased by 3.7-fold, 4.5-fold, and 3.2-fold among Y-oil, Ox, and Ox-Y@NSs-treated cells, respectively (Figure 6). Moreover, the expression levels of the tumor suppressor protein Tp53 tended to increase, something that was observed among all the treated cells; this increase was significant among Y-oil (1.6-fold) and Ox-Y@NSs (2-fold)-treated cells (Figure 6). The Bcl-2 gene is widely known to oppose the Bax activity and to antagonize its action, thus hindering the apoptotic pathway [58]. In the current study, a significant decline in the expression of Bcl2 was observed among the Ox and Ox-Y@NSs-treated cells, whereby it decreased by 34% and 80% compared to untreated cells (Figure 6). Studies have also shown that apoptosis may result from the sequential activation of caspases 9 and 7 in breast cancer cells [59]. Bax has demonstrated the solid potential to activate caspases 9 and 7 during both in vitro and in vivo studies [60]. The Caspase-7 gene expression level was significantly elevated by 1.5-fold and 2.9-fold among Ox and Ox-Y@NSs, respectively (Figure 6). All the described results provide robust evidence for the apoptotic effects of the prepared noisome Ox-Y@NSs, which caused a remarkable increase in the expression levels of the apoptotic markers Bax, Caspase-7, and Tp53, with a noticeable decline in the expression level of the antiapoptotic Bcl2.

### 2.7. Western Blotting

The molecular effects of the three investigated tested compounds (Ox, Y-oil, and Ox-Y@NSs) on the Caspase-7 gene involved in mitochondrial apoptosis have been further evaluated. Western blotting was performed to assess the relative normalized protein expression of the apoptotic protein, Caspase-7, in MDA-MB-231 cancer cells when pre-treated for 48 h with the investigated compounds at the estimated IC50 concentrations. 

**Figure 7:**
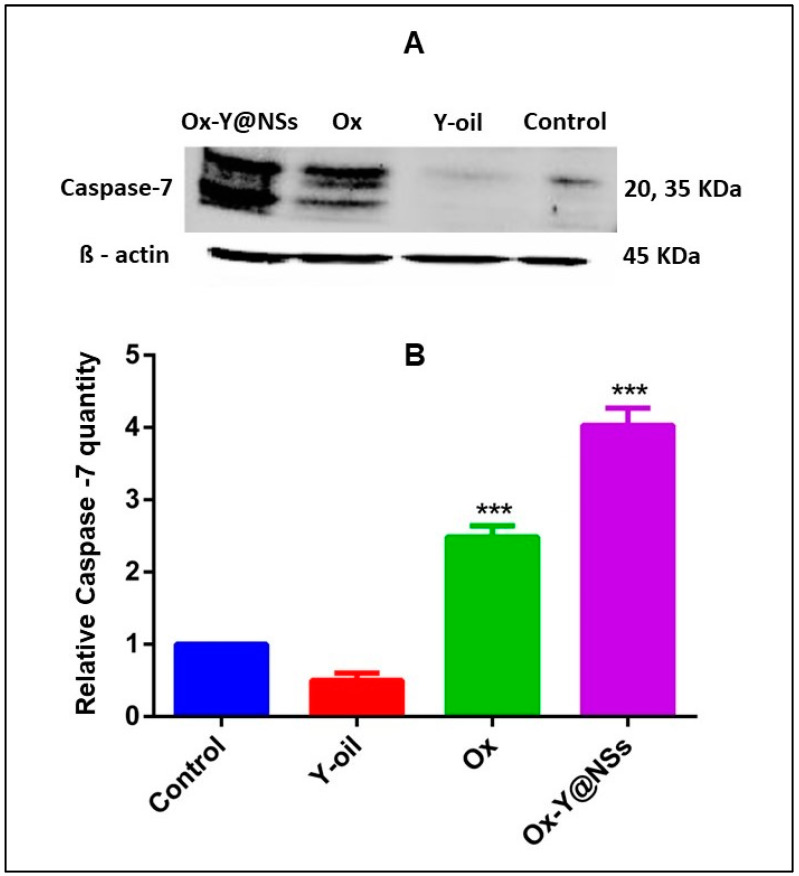
Representative western blot for MDA-MB-231 cellular levels of Caspase-7 in the different treatment groups (**A**). Bar chart representation of the normalized Caspase-7 protein (**B**). Data are normalized using ß-actin and calculated relative to the control (untreated group). Data are presented as mean ± SD (*n* = 3). The symbol *** indicates a significant difference from untreated cells (control) at *p* < 0.001, respectively.

Ox was previously reported to activate Caspase-7 in colorectal and liver cancer cells, thus stimulating apoptosis [61,62]. Herein, a significant elevation in the protein expression of Caspase-7 was observed in MDA-MB-231 cells treated with Ox and Ox-Y@NSs, whereby the treatments led to an approximately 2.5-fold and 4-fold increase, respectively, compared to the control group (Figure 7). This partly explains the potentiated cytotoxic and apoptotic effects of the synthesized Ox-Y@NSs, which makes the designed nanovesicles override the reference drug, Ox, with regard to the cytotoxicity and apoptotic profiles. The results align with the relative gene expression assessed using RT-qPCR, establishing additional evidence for our findings. They also shed light on the ability of Ox-Y@NSs to initiate late apoptosis by stimulating the late apoptotic marker Caspase-7 in MDA-MB-231 cells.

## 3. Materials and Methods

### 3.1. Materials

Sorbitan stearate (Span 60) and polysorbate 60 (tween 60) were purchased from Biosynth Carbosynth, Berkshire, UK. Cholesteryl hemisuccinate (CHEMS) and sodium dodecyl sulfate (SDS) were purchased from Sigma-Aldrich (St. Louis, MO, USA). Chloroform was provided by Merck Company (Darmstadt, Germany). Phosphate-buffer saline (PBS, pH 7.4) and acetate buffer (pH 5.4) were supplied by Lonza Bioscience (Walkersville, MD, USA). The remaining reagents were all of analytical grade. Dulbecco’s Modified Eagle Medium (DMEM) and fetal bovine serum (FBS) were purchased from Gibco (Thermoscientific, Germany). The Penicillin–Streptomycin mixture was obtained from Lonza (Bioscience, Switzerland). 

### 3.2. Methods

#### 3.2.1. Extraction and Identification of Essential Oils from Cananga Odorata Flowers

Y-oil was obtained using an eco-friendly approach via the hydrodistillation of fresh, fully opened Ylang ylang flowers (*Cananga odorata*, forma *genuine*) cultivated in Reunion Island, France. Hydrodistillation was conducted in a Clevenger device, in which 150 g of fresh Ylang ylang flowers were added to 1000 mL of distilled water. The process occurred at 100 °C, then decreased to 70 °C while the cooling system temperature was kept at 4 °C. The distilled Y-oil was stored in tightly closed dark glass bottles at 4 °C for further studies. The constituents of the extracted Y-oil were then identified via GC–MS analysis utilizing Agilent Technologies gas chromatography (7890B) and a mass spectrometer detector (5977B), following our previously reported method [44].

#### 3.2.2. Preparation of the pH-Responsive Niosomal Nanovesicles

The thin film hydration technique was adopted to prepare the blank niosomes, Y-oil-loaded niosomes (Y@NSs), oxaliplatin-loaded niosomes (Ox@NSs and niosomes dual loaded with OX and Y-oil (Ox-Y@NSs), with some modifications [38,63].

Briefly, 120 mmol of surfactants (Tween 60 and Span 60) and cholesteryl hemisuccinate (CHEMS, a pH-responsive lipid) were used in a molar ratio of 1:1:2. The surfactants and CHEMS (with or without Y-oil) were dissolved in a methanol/chloroform mixture (1:2, v/v) in a round-bottom flask. Then, the organic solvents were removed using rotary evaporation under vacuum for 40 min at 58 °C. The formed thin film was then hydrated with phosphate buffer saline (pH of 7.4), with or without Ox, using the rotary evaporator under normal pressure for 60 min at 58 °C, forming multilamellar vesicles. Afterward, the produced suspension was probe sonicated using a Pulse 150 Benchmark ultrasonic homogenizer (NJ, USA) for 2 min to generate unilamellar niosomes. The probe sonication process was conducted by applying the simultaneous on and off mode (every two seconds) to prevent the destruction of the niosomes. The generated unilamellar nanovesicles were kept at room temperature for 60 min and then stored at 4 °C for further studies.

#### 3.2.3. Characterization of the Prepared pH-Responsive Niosomal Nanovesicles

The mean particle sizes, polydispersity index (PDI), and zeta-potential were measured using a Zetasizer Nano (Malvern Instruments, Herrenberg, UK), where all measurements were carried out at 25 °C.

A transmission electron microscope (JEOL-JEM 2100, Musashino, Akishima, Tokyo, Japan), operating at 160 kV, studied the morphological features of the developed niosomal nanovesicles.

#### 3.2.4. Determination of Entrapment Efficiency % (EE) of Y-Oil and Ox in pH-Responsive Niosomal Nanovesicles

The indirect method was utilized to determine the percent of Y-oil and OX entrapped (EE %) in Y@NSs, Ox@NSs, and Ox-Y@NSs. In Brief, an aliquot of each sample (2 mL) was ultracentrifuged at 14,000 rpm for 120 min at 4 °C. Afterward, the supernatant of each sample was separated, ultrafiltrated, and then the unentrapped OX was determined using HPLC, as described in our previously reported study [38]. On the other hand, the unentrapped Y-oil was quantified using UV–Vis spectrophotometry (Peak instruments T-9200, USA) at 290 nm. The *EE* (%) was calculated by employing Equation (1) [36,64,65].

(1) $$EE\;\%=\frac{Initial\;amount\;of\;drug-the\;amount\;of\;unentrapped\;drug\;}{Initial\;amount\;of\;drug}\times 100$$

#### 3.2.5. In Vitro Release Study

The Y-oil and Ox release rates from Y@NSs, Ox@NSs, and Ox-Y@NSs were studied by exploiting the dialysis bag method at two different pH values (pH 7.4 and pH 5.4). Concisely, a dialysis bag (14KD cut off) containing 1 mL of each preparation was immersed in 25 mL of either PBS (pH 7.4) or acetate buffer (pH 5.4) as the release medium. Sodium dodecyl sulfate (SDS, 0.5%) was added to the release medium to increase the hydrophobic drugs’ solubility and decrease the release resistance. Then, the system was located in a shaking incubator (Jeio tech SI-300, SEOUL, KOREA) at 37 ± 0.5 °C and a shaking rate of 130 rpm. At definite time intervals, 1 mL of each sample was retrieved, and the concentration of Ox was quantified via HPLC, as previously described [3]; meanwhile, that of the Y-oil was detected by UV–vis spectrophotometry at 278 nm. The withdrawn sample was immediately replenished with an equal volume of fresh medium to maintain sink conditions. The release % of Y-oil and Ox was estimated using Equation (2).

(2) $$Release\%=\frac{Amount\;of\;released\;drug}{Initial\;amount\;of\;loaded\;drug}\times 100\;$$

#### 3.2.6. Cell Culture

Triple-negative breast cell lines (MDA-MB-231) were obtained from the American type of culture collection (ATCC, Wesel, Germany). The cells were cultured at standard conditions by keeping them in DMEM media and adding 100 mg/mL of streptomycin, 100 units/mL of penicillin, and 10% of heat-inactivated fetal bovine serum in humidified media. The culture plates were placed in a 5% (*v*/*v*) CO_2_ incubator at 37 °C.

#### 3.2.7. Cytotoxicity Assay

SRB assay was utilized to detect the cytotoxicity of six investigated agents. The test compounds were as follows: Blank NSs, Y-oil, Y@NSs, Ox, Ox@NSs, and Ox-Y@NSs. A volume of 100μL cell suspension containing approximately 5000 cells was placed in each well of a 96-well plate and incubated in complete culture media for one complete day. Then, 100μL media of the test compounds was applied to the cells at eight concentrations (0.0001, 0.001, 0.01, 0.1, 1, 10, 100, and 1000 µg/mL). Then, the plates were incubated for 72 h. Directly afterward, the whole media were removed, and a fixation step was performed by adding 150 μL of 10% TCA per well and incubating at 4 °C for 1 h. The TCA solution was then removed, and five sequential wash steps with distilled water were applied to the wells. A volume of 70 μL of SRB solution (0.4% *w*/*v*) was added to each well, and the plates were kept in the darkroom at the typical room temperature (25 °C) for 10 min. This was followed by 3 steps of washing with 1% acetic acid. Then, the plates were left to airdry overnight. Lastly, 150 μL of TRIS (10 mM) was added to dissolve the protein-bound SRB stain in each well, and the absorbance was read at 540 nm using a BMGLABTECH^®^-FLUOstar Omega microplate reader (Ortenberg, Germany).

The investigated compounds’ combination index (CI) was computed using the isobologram equation to determine whether the combination was synergistic, additive, or antagonistic, as shown in Equation (3) [37,48].

(3) $$\mathrm{CI}=\frac{d1}{D1}+\frac{d2}{D2}$$

where ***d***1 and ***d***2 are the concentrations of Y-oil and Ox capable of inducing the same cytotoxic effect when combined in Ox-Y@NSs, while ***D***1 and ***D***2 are the IC50s of Ox@NSs and Y@NSs, respectively. The combination is considered synergistic if CI < 0.8; additive if CI ranges from 0.8 to 1.2; and antagonistic if CI > 1.2 [37,48].

#### 3.2.8. Apoptosis Assay

The Annexin V-FITC apoptosis detection kit (Abcam Inc., Cambridge, UK) and two fluorescent channels flow cytometry were utilized to assess the apoptotic profile of three of the test compounds (Y-oil, Ox, and Ox-Y@NSs), as per the manufacturer’s guidelines. Briefly, MDA-MB-231 cells were treated with the test compounds at their IC50s concentrations for 48 h. The cells (10^5^ cells) were then harvested by trypsinization, and ice-cold PBS (pH 7.4) was applied to the cells in two sequential wash steps. Afterward, a volume of 0.5 mL Annexin V-FITC/PI solution was applied to the cells, and the cells were kept away from light and incubated with this solution for about half an hour. When the staining was complete, cells were assayed for FITC and PI fluorescent signals by the ACEA Novocyte™ flow cytometer (Biosciences Inc., San Diego, CA, USA). The two signal detectors were implemented as follows. The fluorescence wavelength (λex/em 488/530 nm) was kept for FITC, while the fluorescence wavelength (λex/em 535/617 nm) was adjusted for PI staining. An estimate of 12,000 events were acquired per sample. Lastly, the ACEA NovoExpress™ built-in software was implicated for quadrant analysis and the determination of the positive FITC and/or PI cells.

#### 3.2.9. RT-qPCR

##### RNA Extraction

RNA extraction was performed for the cells treated with the three investigated compounds (Y-oil, Ox, and Ox-Y@NSs), as well as for untreated cells (Control), through the utilization of a QIA amp Viral RNA Mini Kit (Qiagen, Hilden, Germany), following the manufacturer’s instructions. Briefly, a volume of 40 μL of RNA solution was isolated from 100 μL of each sample. Then, the Nanodrop Spectrophotometer (A260/280 ratio) was used to assess the exact quantity and quality of the extracted RNA.

#### 3.2.10. cDNA Synthesis

cDNA synthesis was performed for RNA using the ReverAid RT Kit (ThermoFisher Scientific, Waltham, USA). The kit was designed to reverse transcribe 5 μL of RNA using a random hexamer primer, as prescribed by the manufacturer. The reaction components (Table 5) were mixed up well, and cDNA synthesis was performed on the Bio-Rad TM 100 Thermal cycler.

#### 3.2.11. qPCR

The RT-qPCR analysis of the required genes (ß-actin, Bax, Bcl2, Caspase-7, and Tp53) was performed using the Qiagen Quanti Nova SYBR Green PCR Kit (cat # 208052). The primers used are presented in Table 6. The reaction components are clearly demonstrated in Table 7. qPCR was performed using a Rotor-Gene Q-Qiagen Real-time PCR thermal cycler.

#### 3.2.12. Western Blotting

Western blot analysis was performed to assess the relative quantity of Caspase-7protein among the cells treated with the test compounds (Y-oil, Ox, and Ox-Y@NSs). Untreated cells were used as a control, and ß-actin protein was utilized for normalization. The procedures went as follows. At first, the lysis of MDA-MB-231 cells’ pellets was performed using 10 mM of Tris-HCl, 100 mM of NaCl, 0.5% Triton X-100, a pH of 7.6 and an EDTA-free Protease Inhibitor Cocktail. The protein quantity was measured using a Pierce™ BCA Protein Assay Kit (ThermoFisher Scientific). Then, 25 ug of protein from each sample was subjected to sodium dodecyl sulfate-polyacrylamide gel (SDS-PAGE). Western blot analysis was carried out with the primary antibodies described in Table 8 against Caspase-7 and ß-actin. HRP-conjugated secondary antibodies were added, as shown in Table 8. The chemiluminescence reaction was performed using the ECL western blot HRP substrate (Pierce, ThermoFisher Scientific). Finally, the ChemiDoc imaging system of Bio-Rad Co was used in the image acquisition, as well as in the densitometry calculations. 

#### 3.2.13. Statistical Analysis

Statistical analysis was performed using GraphPad Prism 6. All data were reported as the means of triplicate individual runs ± SD. The data with a normal distribution were analyzed using ANOVA with multiple comparisons post hoc test. Regarding the data that did not follow the normal distribution, non-parametrical Kruskal–Wallis, and Mann–Whitney tests were used. *p*-value ≤ 0.05 was considered statistically significant. The symbols ***, **, * refer to *p*-values ≤0.001, ≤0.01 and ≤0.05, respectively. 

## 4. Conclusions

Non-targeted cancer therapies cause severe toxic effects that hinder their clinical applications and can be amended using pH-responsive nanocontainers. In this regard, Ox, and/or Y-oil (extracted from *Cananga odorata* flower) were loaded into smart pH-responsive niosomes based on CHEMS (a pH-sensitive agent) using a thin film technique. All formulations displayed higher cytotoxic activities against the invasive human breast cancer cell lines compared to the free Ox or Y-oil. The niosomes co-loaded with Ox and Y-oil (Ox-Y@NSs) exhibited the lowest IC50 value compared to those loaded with either Ox or Y-oil, suggesting a synergistic anticancer effect. In addition, Ox-Y@NSs treatment resulted in the highest percentages of late apoptotic and necrotic MDA-MB-231 cells. The study deeply investigated apoptosis on the molecular level. The synthesized dually loaded pH-responsive niosomes demonstrated an outstanding ability to upregulate Tp53, Bax, and Caspase-7 and downregulate Bcl-2 gene expression. A protein assay also provided evidence that Ox-Y@NSs increased the relative protein quantity of the late apoptotic marker, Caspase-7. The developed intelligent dually loaded niosomes, Ox-Y@NSs, exhibited strong cytotoxic and apoptotic abilities, demonstrating that they are an optimal candidate for cancer therapy and thus warrant further preclinical and clinical investigations.

## Figures and Tables

**Table 1:** The relative percentage of volatile compounds detected in Y-oil (*Cananga Odorata* Hook. F. and Thomson) via GC–MS measurements (*n* = 3). Results are average of three independent replicates (standard deviation).

Peak No.	Compound Name	RT (min)	RI	Average % (SD)
**Alcohols**				
**1.**	Benzyl alcohol	9.90	1031.9	2.03 (0.51)
**2.**	2-(2-Hydroxypropoxy)-1-propanol	10.16	1044.8	0.72 (0.21)
**3.**	2,2’-oxybis-1-propanol	10.34	1053.1	0.68 (0.04)
**4.**	β-Linalool	12.16	1105.3	9.15 (1.48)
**5.**	Phenylethyl Alcohol	12.58	1115.3	0.22 (0.12)
**6.**	α-Cedrol	28.69	1593.3	2.28 (0.60)
**7.**	Spathulenol	29.09	1616.7	0.14 (0.00)
**8.**	α-Cadinol	30.95	1642.4	0.22 (0.03)
**Total alcohols**				**15.44**
**Esters**				
**9.**	Benzyl formate	11.33	1078.5	0.21 (0.18)
**10.**	Methyl benzoate	11.93	1089.8	1.40 (0.45)
**11.**	Benzyl acetate	14.38	1175.2	10.47 (2.03)
**12.**	Methyl salicylate	15.39	1194.2	0.17 (0.04)
**13.**	Linalyl acetate	17.49	1254.8	3.22 (0.90)
**14.**	Geranyl acetate	21.69	1379	0.39 (0.05)
**15.**	Nopyl acetate	22.98	1427.2	2.71 (0.45)
**16.**	Cinnamyl acetate	23.59	1438.6	0.22 (0.05)
**17.**	Benzyl Benzoate	35.89	1767.3	0.95 (0.08)
**18.**	Farnesyl acetate	38.9	1839.9	0.48 (0.27)
**19.**	Benzyl salicylate	39.64	1862.5	0.30 (0.07)
**Total esters**				**20.52**
**Phthalate esters**				
**20.**	Diethyl Phthalate	28.62	1584.2	29.08 (2.67)
**Total glycerols**				**29.08**
**Monoterpene hydrocarbons**				
**21.**	Limonene	9.68	1029.5	0.61 (0.04)
**22.**	Allo-Ocimene	13.12	1129.6	0.21 (0.03)
**23.**	(4E,6Z)-Allo-Ocimene (Neo-allo-ocimene)	13.55	1140.2	0.16 (0.06)
**Total monoterpene hydrocarbons**				**0.98**
**Phenols/ethers**				
**24.**	*p*-Methyl anisole	9.41	978.2	1.47 (0.12)
**25.**	Bis(2-hydroxypropyl) ether	9.55	994.8	0.48 (0.01)
**26.**	Estragole	15.52	1199.6	0.51 (0.03)
**27.**	Eugenol	20.85	1358.5	0.96 (0.07)
**28.**	Methyleugenol	22.37	1408.8	0.60 (0.15)
**Total phenols/ethers**				**4.02**
**Sesquiterpene hydrocarbons**				
**29.**	α-Cubebene	20.59	1352	0.05 (0.03)
**30.**	α-Copaene	21.45	1375.2	0.59 (0.03)
**31.**	β-Elemene	21.97	1391.6	0.17 (0.02)
**32.**	Cyperene	22.2	1403.5	0.94 (0.26)
**33.**	α-Gurjunene	22.57	1410.3	11.04 (2.79)
**34.**	α-Cedrene	22.62	1413.3	0.80 (0.12)
**35.**	β-Caryophyllene	22.84	1425.5	0.88 (0.27)
**36.**	Thujopsene	23.2	1429.5	6.83 (1.22)
**37.**	Humulene	23.9	1444.7	0.21 (0.00)
**38.**	Aromandendrene	24.13	1454.5	2.45 (0.08)
**39.**	γ-Gurjunene	24.48	1471.4	1.08 (0.29)
**40.**	γ-Muurolene	24.61	1478.3	0.35 (0.07)
**41.**	Germacrene D	24.74	1481	0.22 (0.03)
**42.**	Ledene	25.17	1492.2	0.33 (0.05)
**43.**	α-Muurolene	25.32	1498.1	0.15 (0.01)
**44.**	Cuparene	25.49	1504.3	0.23 (0.15)
**45.**	(E,E)-α-Farnesene	25.54	1506.9	0.13 (0.04)
**46.**	γ-Cadinene	25.74	1509.2	0.13 (0.03)
**47.**	δ-Cadinene	26.02	1525.8	0.75 (0.06)
**Total sesquiterpene hydrocarbons**				**27.33**
**Others**				
**48.**	Piperonal	20.03	1330.8	0.21 (0.03)
**49.**	1,2-Diacetin	20.68	1356.1	0.11 (0.00)
**50.**	Caryophyllene oxide	27.97	1576.4	0.15 (0.11)
**51.**	Unidentified	37.98	1812.4	0.20 (0.02)
**52.**	Benzylidene camphor	41.65	1914.5	1.96 (0.27)
**Total volatiles**				**100**

**Table 2:** The mean particle size, PDI, and zeta-potential of different pH-responsive niosomal nanovesicles. Data are presented as mean ± SD; *n* = 3.

Samples	Mean Particle Size (nm)	PDI	Zeta-Potential (mV)	EE (%)	
				Y-oil	Ox
**Plain NSs**	94.16 ± 19.0	0.12 ± 0.03	−12.37 ± 1.7	-	-
**Y@NSs**	129.72 ± 15.5	0.16 ± 0.02	−14.65 ± 1.5	93.1 ± 6.1	-
**Ox@NSs**	126.93 ± 12.5	0.15 ± 0.02	−10.53 ± 1.1	-	83.9 ± 4.3
**Ox-Y@NSs**	159.34 ± 16.9	0.19 ± 0.05	−13.31 ± 1.9	91.6 ± 7.5	81.1 ± 5.8

**Table 3:** IC_50_ values of different samples obtained via the cytotoxicity analysis against triple-negative breast cancer (MDA-MB231) after 72 h treatment with the corresponding agent.

Sample	IC50 on MDA-MB231 Cell Line ^#^ (µg/mL)
**Blank NSs**	184.63
**Y-oil**	29.01
**Y@NSs**	18.39
**Ox**	0.05
**Ox@NSs**	0.006
**Ox-Y@NSs**	0.0002

*^#^* IC_50_ of the test compounds was calculated using Sigma Plot 12.0 software.

**Table 4:** Apoptosis assay of MDAMB-231 cells after treatment with Ox, Y-Oil, and Ox-Y@NSs at the corresponding IC_50_ values for 48 h.

Apoptotic Stage	Percent Cell Population			
	Control	Ox	Y-Oil	Ox-Y@NSs
**Necrosis (Q2-1)**	0.58 ± 0.13	* 1.93 ± 0.59	** 2.13 ± 0.19	** 5.06 ± 1.14
**Late apoptosis (Q2-2)**	1.29 ± 0.24	*** 7.33 ± 0.7	* 2.3 ± 0.32	*** 8.38 ± 0.83
**Viable cells (Q2-3)**	98.02 ± 0.07	*** 90.29 ± 1.29	** 95.45 ± 0.42	*** 86.34 ± 1.49
**Early apoptosis (Q2-4)**	0.11 ± 0.02	*** 0.44 ± 0.03	0.11 ± 0.06	* 0.22 ± 0.06

The provided percent is the mean of triplicate independent runs ± standard deviation. *p*-value ≤ 0.05 is considered statistically significant compared to the control. *, **, and *** refer to *p*-values ≤ 0.05, ≤0.01, and ≤0.001, respectively.

**Table 5:** cDNA synthesis reaction components.

Reaction Component	Amount
**RNA**	1 µg
**Random Hexamers**	2.5 µL (from 100 µM stock)
**5X Enzyme Buffer**	5 µL
**dNTP**	1.7 µL (from 40 mM stock)
**AMV-RT (20U)**	2 µL
**RNase free water**	XX
**Total volume**	25 µL

**Table 6:** Primersused in RT-qPCR.

Primer ID	Primer Sequence (5′-3′)	Company
**β-actin F**	CACCATTGGCAATGAGCGGTTC	Metabion International AG
**β-actin R**	AGGTCTTTGCGGATGTCCACGT	
**Bax F**	TCAGGATGCGTCCACCAAGAAG	
**Bax R**	TGTGTCCACGGCGGCAATCATC	
**Bcl-2 F**	ATCGCCCTGTGGATGACTGAGT	
**Bcl-2 R**	GCCAGGAGAAATCAAACAGAGGC	
**Caspase-7 F**	CGGAACAGACAAAGATGCCGAG	
**Caspase-7 R**	AGGCGGCATTTGTATGGTCCTC	
**TP53 F**	CCTCAGCATCTTATCCGAGTGG	
**TP53 R**	TGGATGGTGGTACAGTCAGAGC	

**Table 7:** qPCR reaction components.

qPCR Reaction Component	Amount
**Qiagen Master Mix 2X**	10 µL
**F primer (10 µM)**	1 µL
**R primer (10 µM)**	1 µL
**cDNA**	100 ng
**Complete with RNase free H_2_O**	X
**Total volume**	20 µL

**Table 8:** Antibodies used for Western blot analysis.

Ab	Species	Dilution Used	Catalog Number	Company
**Caspase-7**	Rabbit	1:1000	#9492T	Cell Signaling
**B-actin**	Rabbit	1:1000	#4970	
**Anti-rabbit IgG, HRP-linked**	Goat	1:3000	#7074P2	

## Data Availability

Data is contained within the article and supplementary material.

## Supplementary Materials

The supporting information can be downloaded at: https://www.mdpi.com/article/10.3390/ijms24098392/s1.
