# Supplementary material for: Co-Delivery of Ylang Ylang Oil of Cananga odorata and Oxaliplatin Using Intelligent pH-Sensitive Lipid-Based Nanovesicles for the Effective Treatment of Triple-Negative Breast Cancer
Source: Int J Mol Sci. 2023 May 7;24(9):8392. doi: 10.3390/ijms24098392 (PMC10179110; doi:10.3390/ijms24098392)
Supplement: Supplementary file 1 [file ijms-24-08392-s001.zip › ijms-2374398-supplementary.pdf]

Co-Delivery of Ylang Ylang Oil of *Cananga odorata* and Oxaliplatin Using Intelligent pH-Sensitive Lipid-Based Nanovesicles for the Effective Treatment of Triple-Negative Breast Cancer

Nada K. Sedky [1,†], Nour M. Abdel-Kader [1,2,†], Marwa Y. Issa [3], Manal M. M. Abdelhady [4], Samir N. Shamma [5], Udo Bakowsky [6,*] and Sherif Ashraf Fahmy [7,*]

[1] Department of Biochemistry, School of Life and Medical Sciences, University of Hertfordshire Hosted by Global Academic Foundation, R5 New Garden City, New Administrative Capital, Cairo 11835, Egypt
[2] Department of Biochemistry, Faculty of Science, Ain Shams University, Cairo 11566, Egypt
[3] Department of Pharmacognosy, Faculty of Pharmacy, Cairo University, Kasr El-Aini Street, Cairo 11562, Egypt
[4] Clinical Pharmacy Department, Faculty of Pharmacy, Badr University, Cairo 11829, Egypt
[5] Institute of Global Health and Human Ecology, School of Sciences & Engineering, The American University in Cairo, AUC Avenue, P.O. Box 74, New Cairo 11835, Egypt
[6] Department of Pharmaceutics and Biopharmaceutics, University of Marburg, Robert-Koch-Str. 4, 35037 Marburg, Germany
[7] Department of Chemistry, School of Life and Medical Sciences, University of Hertfordshire Hosted by Global Academic Foundation, R5 New Garden City, New Administrative Capital, Cairo 11835, Egypt
* Correspondence: ubakowsky@aol.com (U.B.); s.fahmy@herts.ac.uk or sheriffahmy@aucegypt.edu (S.A.F.); Tel.: +49-(0)-6421-28-2-58-84 (U.B.); +20-1222613344 (S.A.F.)
† These authors contributed equally to this work.

Supplementary Materials

Phthalate esters were the major detected volatile class in Y-oil, encompassing diethyl pthalate as the only component that reached ca 29.08 %.

Sesquiterpene hydrocarbons comprised the second most abundant class of volatiles in Y-oil, accounting for 27.33 % of its total volatiles. α-Gurjunene (peak **33**) was the major detected sesquiterpene hydrocarbon and the second most abundant volatile component in Y-oil, accounting for ca. 11.04 % of its total essential oil composition, where thujopsene, aromandendrene, and γ-gurjunene (peaks **36, 38** and **39**) amounting for ca. 6.83, 2.45 and 1.08 of the total volatile components in Y-oil, respectively. Additional minor sesquiterpene hydrocarbons were detected in lesser amounts.

Esters were the third dominant volatile class (20.52 %), represented by benzyl acetate as the major ester (10.47 %, peak **11**), then linalyl acetate (3.22 %, peak **13**), followed by Nopyl acetate (2.71 %, peak **15**), and methyl benzoate (1.40 %, peak **10**). Other esters found in fewer amounts were benzyl benzoate, farnesyl acetate, geranyl acetate, benzyl salicylate, cinnamyl acetate, benzyl formate, and methyl salicylate.

Alcohols constituted the fourth abundant volatiles class, accounting for 15.44 %. β-linalool was the major alcohol detected in Y-oil, accounting for ca. 9.15 % (peak **4**), followed by α-Cedrol (2.28 %, peak **6**) and Benzyl alcohol (2.03, peak **1**), in addition to other minor detected alcohols.

Phenol/ethers was the fifth group in order of volatile content of Y-oil (4.02 %) comprising p-methyl anisole, eugenol, methyleugenol, estragole, and bis(2-hydroxypropyl) ether at concentrations 1.47, 0.96, 0.6, 0.51 and 0.48 of the total volatile content of Y, respectively.

Three monoterpene hydrocarbons only were detected in Y-oil, accounting for about 0.98 %, namely, limonene, allo-Ocimene, and neo-allo-ocimene at concentrations 0.61, 0.21, and 0.16 %, respectively, of the total volatile content of Y-oil.

In addition, four other volatile classes, viz. ketones, aldehydes, oxides, and glycerols, each comprising only one constituent in minor amounts, namely, benzylidene camphor, piperonal, caryophyllene oxide and 1,2-diacetin, respectively.
